# Isolation and Biochemical Characterization of Rubelase, a Non-Hemorrhagic Elastase from *Crotalus ruber ruber* (Red Rattlesnake) Venom

**DOI:** 10.3390/toxins3070900

**Published:** 2011-07-19

**Authors:** Yumiko Komori, Kaname Sakai, Katsuyoshi Masuda, and Toshiaki Nikai

**Affiliations:** 1 Department of Microbiology, Faculty of Pharmacy, Meijo University, 150 Yagotoyama, Tenpaku, Nagoya 468-8503, Japan; Email: kaname_sakai@yahoo.co.jp (K.S.); nikai@meijo-u.ac.jp (T.N.); 2 Suntory Institute for Bioorganic Research, Shimamoto-cho, Mishima-gun, Osaka 618-8503, Japan; Email: Katsuyoshi_Masuda@suntory.co.jp

**Keywords:** *Crotalus ruber ruber* toxin, elastase, amino acid sequence, cytotoxicity

## Abstract

A novel non-hemorrhagic basic metalloprotease, rubelase, was isolated from the venom of *Crotalus ruber ruber*. Rubelase hydrolyzes succinyl-L-alanyl-L-alanyl-L-alanyl *p*-nitroanilide (STANA), a specific substrate for elastase, and the hydrolytic activity was inhibited by chelating agents. It also hydrolyzes collagen and fibrinogen. However, hemorrhagic activity was not observed. By ESI/Q-TOF and MALDI/TOF mass spectrometry combined with Edman sequencing procedure, the molecular mass of rubelase was determined to be 23,266 Da. Although its primary structure was similar to rubelysin (HT-2), a hemorrhagic metalloprotease isolated from the same snake venom, the circumstances surrounding putative zinc binding domain HEXXHXXGXXH were found to be different when the three-dimensional computer models of both metalloproteases were compared. The cytotoxic effects of rubelase and rubelysin on cultured endothelial and smooth muscle cells were also different, indicating that the substitution of several amino acid residues causes the changes of active-site conformation and cell preference.

## 1. Introduction

Many kinds of enzymes and biologically active peptides are present in snake venoms. Hemorrhage is one of the characteristic symptoms associated with Viperidae snake envenomation. In case of severe envenomation, hemorrhage can take place in many internal organs [1,2]. Several investigators have reported the purification, characterization, and structure of hemorrhagic principles from the venom of *Protobothrops flavoviridis* [3], *Protobothrops mucrosquamatus* [4], *Deinagkistrodon acutus* [5], *Crotalus atrox* [6,7], *Crotalus ruber ruber* [8] and *Agkistrodon bilineatus* [9]. These investigators demonstrated that these hemorrhagic toxins have proteolytic activity capable of hydrolyzing the oxidized insulin B chain, fibrinogen, glucagon, and hide powder azure. The primary structures of various hemorrhagic toxins have been determined [10,11,12,13,14,15,16,17] and crystal structures of some low molecular weight toxins have been reported [18,19,20,21,22,23]. Recently, these toxins have been categorized as snake venom metalloproteases (SVMPs), and classified as members of ADAMs proteins [24,25]. On the other hand, the presence of elastase in snake venoms has been discussed for many years, but the conclusion has not been reached. Early study on the venom of red diamond rattlesnake (*C. r. ruber*) indicated that two major size classes of proteases are present in the venom [26]. However, only hemorrhagic toxins have been well studied. In this paper, we report the isolation and biochemical characterization of novel non-hemorrhagic elastase from the venom of *Crotalus r. ruber*.

## 2. Materials and Methods

Lyophilized crude venom was purchased from Miami Serpentarium Laboratories (U.S.A.). TOYOPEARL^TM^ HW-50 was obtained from Tosoh Co., Ltd., Japan, and CM-cellulose was from Whatman Laboratory (England). Succinyl-L-alanyl-L-alanyl-L-alanine *p*-nitroanilide (STANA), glutaryl-L-alanyl-L-alanyl-L-prolyl-L-leucine *p*-nitroanilide (GAAPLNA), and succinyl-L-alanyl-L-prolyl-L-alanine *p*-nitroanilide (SAPANA) were the products of Peptide Institute Inc. (Japan). Casein and bovine fibrinogen were supplied by Merck Laboratories and Daiichi Pure Chemicals (Japan), respectively. Hide powder azure, azoalbumin, and azo-dye impregnated collagen were purchased from Sigma Chemical Co. Lysyl endopeptidase and clostripain were the products of Roche. CPKII-test Wako^TM^ for creatine phosphokinase activity was supplied by Wako Pure Chemical Industries, Ltd. (Japan). Cryo-preserved human umbilical vein endothelial cells (HUVEC), human pulmonary artery endothelial cells (HPAEC), human aortic smooth muscle cells (HASMC), and their respective cell culture medium were obtained from Kurabo (Japan). Cell counting kit-8^TM^ and CellTracker^TM^ green fluorescent probe CMFDA (5-chloromethylfluororescein diacetate) were supplied by Dojindo laboratories (Japan) and Lonza (USA), respectively.

### 2.1. Isolation and Biochemical Properties

**Figure 1:**
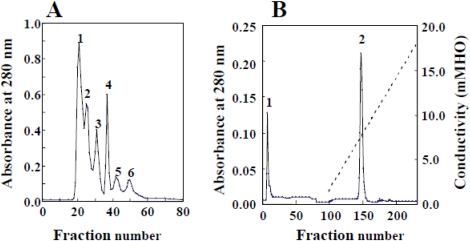
Isolation of rubelase from *Crotalus r. ruber* venom by chromatography. (**A**) HW-50 gel filtration. *Crotalus r. ruber* crude venom (69 mg) was applied to a column (1.5 × 100 cm) equilibrated with 0.01 M Tris-HCl buffer (pH 7.2) containing 0.01 M NaCl. Fractions of 3.0 mL were collected at a flow rate of 10.8 mL/h; (**B**) CM-cellulose column chromatography. The enzyme (fraction 6) was applied to a column (1.5 × 45 cm) equilibrated with the same buffer, and eluted with a linear gradient from 0.01 to 0.5 M NaCl.

Rubelase was isolated from crude venom by HW-50 gel filtration and CM-cellulose cation-exchange column chromatography (see Figure 1). The molecular weight was determined by SDS-polyacrylamide gel electrophoresis and nano ESI-MS using a Micromass Q-TOF mass spectrometer and MassLynx data acquisition. The mass spectrometer was operated in the positive-ion mode. Purified sample was extensively dialyzed against water, lyophilized and then dissolved at a protein concentration of 10 μM in a solution containing equal amounts of acetonitrile and 0.2% formic acid. Two microliters of the sample solution was loaded into a nanoflow tip. A flow rate of about 50 nL/min into the analyzer was produced as a result of a potential of 1.2 kV applied to the nanoflow tip in the ion source. The cone voltage was set to 50 V. The spectra were averaged, smoothed, centroided and deconvoluted.

### 2.2. Enzyme Activities and Pharmacological Activities

Elastase activity was assayed by the method of Hasegawa *et al.* [27] using STANA as the substrate. Proteolytic activity was measured by the method of Murata *et al.* [28] using casein as the substrate, and fibrinogenolytic activity was determined by the method of Ouyang and Teng [29]. Other enzyme assays were carried out as follows: arginine ester hydrolytic activity by the method of Roberts [30]; hide powder azure hydrolytic activity by the method of Rinderknecht [31]; azoalbumin hydrolytic activity by the method of Charney and Tomarelli [32]; azocollagen hydrolytic activity by the method of Moore [33]. One unit of STANA hydrolase activity was defined as the amount of enzyme that hydrolyzed 1 µM of substrate per min. The units of other enzyme activities were obtained by means of the following formula: units/mg = (absorbance change per min) × 2/mg of protein. Hemorrhagic activity was determined by the method of Bjarnason and Tu [6], and serum creatine phosphokinase activity was measured by the method based on the procedure of Tanzer and Gilvarg [34].

### 2.3. Determination of Primary Structure and Computer Modeling

Rubelase was enzymatically digested with clostripain and lysyl endopeptidase. The digested fragments were also obtained by autoproteolysis, which occurs when rubelase is incubated with EGTA at a molar ratio of 1:10. The fragments were analyzed by the combination of Edman degradation method and MALDI/TOF mass spectrometry. Applied Biosystems 491 protein sequencer and Model 610A PTH analyzer were used for the sequence analysis in accordance with the manufacturer’s instructions. The molecular mass of digested fragments was also confirmed by using Voyager^TM^ (Applied Biosystems). Lyophilized fragments were dissolved at a concentration of 10 µM in 0.1% trifluoroacetic acid (TFA) and mixed with matrix (10 mg/mL of α-cyano-4 hydroxy cinnamic acid in 70% acetonitrile cont. 0.1% TFA). The sample mixture was then applied onto a sample plate and analyzed. MOE^TM^ (a molecular simulation and modeling software; purchased from Chemical Computing Group Inc.) was used for construction of protein models.

### 2.4. Toxicity Test on Cultured Cells

Frozen human endothelial cells (HUVEC and HPAEC) and aortic smooth muscle cells (HASMC) were cultured and maintained in the appropriate medium according to the method of the suppliers’ instructions. For bioassays, cells were seeded at a density of 1.5 × 10^4^ cells/well in 0.1 mL of medium in 96-multiwell plates. Samples were diluted in saline and then added to the cells. After 18 h, cell densities were determined by the colorimetric method using a cell counting kit-8 that was based on the tetrazolium salt/formazan system [35]. Cell staining was performed against the adherent cells on glass-plate dishes. Fluorescent probe CMFDA (0.5 µM in serum-free medium) was used according to the manufacturer’s manual, and the viable cells were observed under a fluorescence microscope.

## 3. Results and Discussion

### 3.1. Isolation and Properties

Crude venom was fractionated using HW-50 column and elastase activity was found in fraction 6 (Figure 1A). The fraction was further purified using CM-cellulose column chromatography and a homologous preparation was found in fraction 2 (Figure 1B). The purified enzyme was named rubelase and the yield of rubelase from 69 mg crude venom was found to be approximately 5.3 mg.

When STANA, GAAPLNA and SAPANA were used as elastase substrate, rubelase preferentially hydrolyzed STANA (4.22 units/mg). Rubelase also possessed hydrolytic activities on hide powder azure (1.68 units/mg), azocollagen (1.18 units/mg) and azoalbumin (0.37 units/mg), and fibrinogenolytic activity (Aα and Bβ chains were hydrolyzed without forming fibrin clot). However, casein and arginine ester hydrolytic activities were not observed. Hemorrhagic activity was not detected when the purified enzyme (12 µg: approximately 40-times higher dose of minimum hemorrhagic dose of rubelysin) was injected *s.c.* into mice. Smooth muscle-damaging activity on mice, which was demonstrated as creatine phosphokinase activity in serum, was not detected, either.

Rubelase was thermolabile and the elastase activity was completely lost after incubation for 10 min at 60 °C. The chelating reagents such as EDTA and EGTA also affected elastase activity at final concentrations of 0.1–0.5 mM.

**Figure 2:**
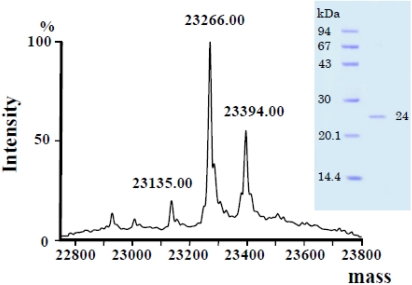
ESI/Q-TOF mass spectra of rubelase from *Crotalus r. ruber* venom. SDS-polyacrylamide gel electrophoresis (*insert*).

The isoelectric point was determined to be 9.2 by electrophoresis. The molecular mass of rubelase determined by SDS-PAGE was found to be 24,000 Da, while the ESI/Q-TOF mass method gave a molecular weight of 23,266 Da (Figure 2). The peak of 23,394 Da found in the macromolecule area indicates that there may be a macromolecule corresponding to rubelase with one residue, such as Lys and Glu, attached to its *C*-terminus. Since this point could be clarified by determining a complete gene sequence, analysis will be attempted if the venom glands of *Crotalus r. ruber* are obtained.

### 3.2. Primary Structure

**Figure 3:**
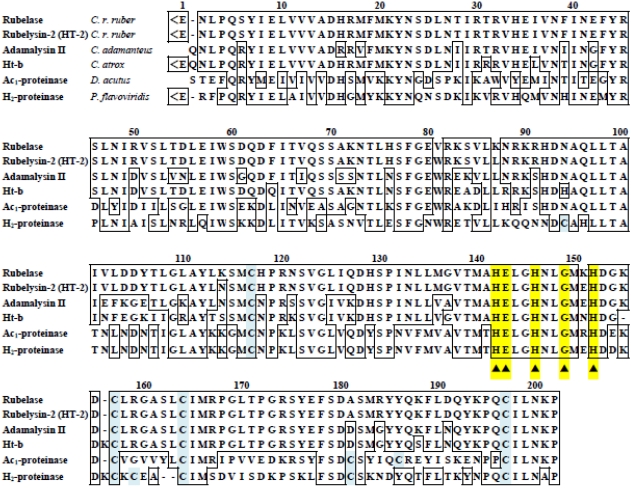
Comparison of the amino acid sequence of rubelase with several low molecular weight metalloproteinases from snake venoms. <E denotes L-pyroglutamic acid. The putative zinc binding ligand and the catalytic domain are indicated by ▲.

The enzymatically cleaved fragments of rubelase by lysyl endopeptidase and clostripain were subjected to Edman sequencing analysis and MALDI/TOF mass spectrometry. The fragments produced by autoproteolysis of rubelase were also analyzed. Although the *N*-terminal amino acid of rubelase could not be detected by a direct sequence analysis of the intact enzyme, analysis of mass spectra of fragments revealed that it was L-pyroglutamic acid. By combining all the data, the complete amino acid sequence of rubelase was determined (Figure 3); rubelase is composed of 202 amino acids. The molecular mass of rubelase based on the amino acid sequence (23267.73 Da) was almost identical to the molecular mass (23,266 Da) obtained by ESI/Q-TOF mass spectra.

### 3.3. Comparison of Rubelase and Rubelysin (HT-2)

The amino acid sequence of rubelase is extremely homologous to rubelysin, a hemorrhagic metalloproteinase previously isolated from *Crotalus r. ruber* venom and named HT-2 [8,13]. The amino acid residues of rubelysin (HT-2) at the position of 81 (Trp), 87 (Leu), 114 (Asn), and 151 (Glu) are substituted for Val, Lys, Lys, and Lys in rubelase, respectively. Rubelysin possesses caseinolytic activity and its minimum hemorrhagic dose (M.H.D) has been determined to be 0.27 µg, whereas rubelase is a non-hemorrhagic protein and does not hydrolyze casein. Since the putative zinc binding domain HEXXHXXGXXH in both enzymes is completely conserved, substitutions of four amino acid residues might cause the difference of substrate preference and biological activity. 

### 3.4. Comparison of Rubelase with the Low Molecular Weight Metalloproteinases

Various low molecular weight metalloproteinases were purified from snake venoms [6,13,36,37,38]. The sequence alignment shown in Figure 3 indicates that rubelase is a member of family of snake venom metalloproteinases (SVMPs). Of these enzymes, adamalysin II (EC 3.4.24.46) isolated from the venom of *Crotalus adamanteus* and H_2_-proteinase from the venom of *Protobothrops flavoviridis* are basic enzymes which have been shown not to exhibit significant hemorrhagic activity [36,38]. Since these properties are similar to rubelase, a significant difference in the caseinolytic activity exists between rubelase and these non-hemorrhagic metalloproteinases.

### 3.5. Toxicity on Cultured Cells

**Figure 4:**
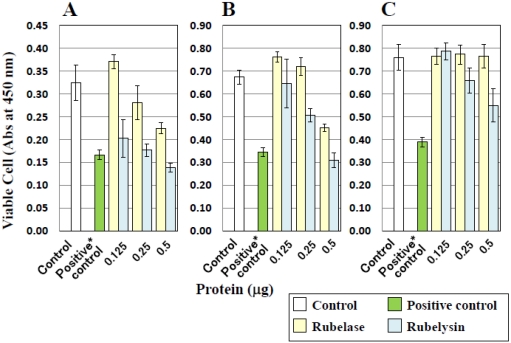
Cytotoxic effects of rubelase and rubelysin on cultured cells. (**A**) HUVEC: human umbilical vein endothelial cells; (**B**) HPAEC: human pulmonary artery endothelial cells; (**C**) HASMC: human aortic smooth muscle cells. Rubelase and rubelysin were added to the cells at various concentrations. After incubation for 18 h, viable cells were counted using the colorimetric method. The results shown represent the average of five experiments. The absorbance of cultured cells incubated with saline or crude venom (10 µg) was defined as control and (cytotoxic) positive control *, respectively.

**Figure 5:**
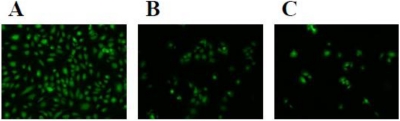
Fluorescence micrographs of HPAEC (×60) after incubation with rubelase and rubelysin. Control cells (**A**); and the cells incubated with rubelase (**B**); and rubelysin (**C**).

The effect of rubelase on cultured cells was investigated and compared with that of rubelysin. Figure 4 shows the changes in cell numbers after incubation with samples. Compared with control cells (white bars), viable HUVEC, HPAEC and HASMC numbers clearly decreased after addition of rubelysin, a hemorrhagic metalloproteinase (light blue bars). When 0.5 µg of rubelysin was added to HUVEC and HPAEC (Figure 4A,B), the number of viable cells decreased to similar levels as seen in the positive control (a cytotoxic control measured by using crude venom: green bars). On the other hand, rubelase possessed cytotoxicity only on HUVEC and HPAEC (Figure 4A,B: light yellow bars), and the effects were relatively weak as compared with rubelysin. The cytotoxic effects of rubelase and rubelysin on endothelial cells (HPAEC) were visualized as the cell density observed under a fluorescence microscope (Figure 5). The viability of HASMC was not affected by rubelase (Figure 4C). These results indicate that rubelase does not inflict an injury on smooth muscle cells which compose blood vessels and tissues. Therefore, rubelase may cause neither hemorrhage nor muscular damage. The muscle damaging activity measured as serum CPK levels also supports this speculation. However, although rubelase is non-hemorrhagic in nature, it clearly possesses cytotoxicity on endothelial cells (Figure 5C) when directly in contact with cells. Rubelase might exhibit its toxicity only when it enters directly into the bloodstream by envenomation.

### 3.6. Molecular Modeling of Rubelase

**Figure 6:**
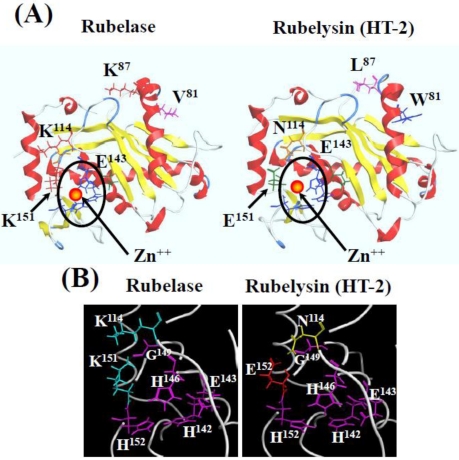
Molecular models of rubelase and rubelysin with the zinc binding site and substituted amino acid residues (**A**); and views of the surrounding structure of the zinc binding site (**B**).

For molecular modeling of rubelase and rubelysin, the information on adamalysin II obtained from the Protein Data Bank (PDB) was referred to, since this protein is the most homologous to these enzymes. Figure 6A shows the protein models of rubelase and rubelysin with the positions of substituted amino acid residues and zinc binding domain. Comparing with rubelysin, the environment surrounding the zinc binding domain (His-Glu-Xxx-Xxx-His-Xxx-Xxx-Gly-Xxx-Xxx-His) of rubelase seem to be basic because of the substitutions of Asn (114) to Lys and Glu (151) to Lys (Figure 6B). These changes may cause the distortion of the zinc binding site in the active pocket, resulting in the decrease in the substrate recognition ability of rubelase against large substrates (e.g., casein).

## 4. Conclusions

A novel non-hemorrhagic metalloprotease, rubelase, hydrolyzes a specific substrate for elastase, and possesses proteolytic activity on collagen and fibrinogen. Its primary structure is similar to the hemorrhagic metalloprotease, rubelysin. However, the cytotoxic effects of rubelase on cultured smooth muscle cells were relatively weak. The three-dimensional computer models of rubelase and rubelysin indicated that the circumstances surrounding zinc binding domain HEXXHXXGXXH were different. The substitution of several amino acid residues might cause the different cell preference and affect cytotoxicity and hemorrhagic activity. Hereafter, the mechanism of unique action of rubelase on vascular endothelial cells should be assessed, also taking the possibility of apoptosis inductive action into consideration. Furthermore, if the venom glands of *Crotalus r. ruber* are obtained, analysis of the gene sequences that code for rubelase and rubelysin will be attempted.
